# Advances in Functional Foods: Using Double Emulsion Gels to Deliver CBD and Probiotics and to Modulate Human Gut Microbial Communities

**DOI:** 10.3390/nu18030367

**Published:** 2026-01-23

**Authors:** Sigita Jeznienė, Ina Jasutienė, Milda Keršienė, Rita Bandariavičiūtė, Laurita Varnaitė-Kapočė, Ieva Bartkuvienė, Vida Audra Budrienė, Arūnas Jonušas, Daiva Leskauskaitė, Aušra Šipailienė

**Affiliations:** 1 Department of Food Science and Technology, Faculty of Chemical Technology, Kaunas University of Technology, Radvilėnų Av. 19, LT-50254 Kaunas, Lithuania; ina.jasutiene@ktu.lt (I.J.); milda.kersiene@ktu.lt (M.K.); rita.bandariaviciute@ktu.lt (R.B.); laurita.varnaite-kapoce@ktu.lt (L.V.-K.); ieva.bartkuviene@ktu.lt (I.B.); daiva.leskauskaite@ktu.lt (D.L.); ausra.sipailiene@ktu.lt (A.Š.); 2 JVC Biosyyd, Vokiečių Str. 161, LT-45273 Kaunas, Lithuania; vb@biosyyd.com (V.A.B.); aj@biosyyd.com (A.J.)

**Keywords:** probiotics, cannabidiol, encapsulation, double emulsion gel, delivery

## Abstract

**Background/Objectives:** This study examines the application of the novel double emulsion gel system for the delivery and release of encapsulated cannabidiol (CBD) and the probiotic strain *Lactiplantibacillus plantarum* DSM 24624. **Methods**: During a six-week experimental period comprising stabilization, treatment, and wash-out phases, the dynamic Simulator of the Human Intestinal Microbial Ecosystem (SHIME^®^) model was employed to assess a system. The evaluation focused on the delivery of CBD and probiotics, as well as the system’s effects on microbial composition, diversity, and metabolic activity throughout the digestion process using 16S rRNA gene sequencing and digital PCR methods. **Results**: Microbial community analysis revealed significant shifts in both mucosal and luminal microbiota following supplementation. The treatment increased beneficial bacterial families such as *Lachnospiraceae* and *Clostridiaceae*, demonstrated effective delivery, release, and persistence of the probiotic *L. plantarum*, as well as enhanced butyrate and lactate production. Diversity analyses highlighted a transient rise in alpha diversity within the mucin layer and a decrease in the lumen, with significant changes in beta diversity across experimental phases. **Conclusions**: Findings suggest that double emulsion gel can be employed for the delivery of probiotics and CBD to the gastrointestinal tract. In addition, an innovative CBD-probiotic formulation can modulate gut microbiota composition and metabolic activity, suggesting its potential as a functional food innovation for intestinal health. However, the results are based on an in vitro model, which lacks the complexity of the human host environment, and further clinical studies are necessary to confirm the biological relevance and therapeutic potential of such delivery systems for gastrointestinal health.

## 1. Introduction

*Cannabis* is obtained from *Cannabis sativa*, an annual herbaceous plant, and has been employed for centuries as an alternative medicinal agent in numerous countries [1]. *Cannabis* is widely recognized for its capacity to produce the psychoactive phytocannabinoid tetrahydrocannabinol (THC). Nevertheless, THC is not the sole phytocannabinoid synthesized by *Cannabis*, nor the only one with biological activity [2]. Among these compounds, cannabidiol (CBD) has garnered considerable attention due to its substantial therapeutic potential. Cannabidiol (CBD) exhibits a broad range of pharmacological activities, including antipsychotic, anxiolytic, antiemetic, anticancer, anti-inflammatory, neuroprotective, antiviral, and antioxidant effects. Additionally, it has been recognized for its role in pain management [3,4]. Importantly, clinical studies conducted in humans have revealed that CBD, even when administered in high oral doses, does not produce tetrahydrocannabinol-like effects, the primary psychoactive compound in cannabis [5,6,7]. Consequently, CBD is classified as non-psychotropic, which has contributed to its increasing exploration and utilization within the human health field. Ongoing clinical trials are further investigating the efficacy of CBD for a variety of conditions. These include studies focused on psychological disorders (NCT05457465; NCT04978428; and NCT05649059), drug-resistant epilepsy (NCT02660255), and pain management (NCT05020028) [8]. The increasing interest in CBD for therapeutic applications has been supported by advances in plant breeding, which have enabled the cultivation of hemp varieties of *Cannabis sativa*. These selectively bred plants produce high concentrations of CBD while keeping the THC content below 0.3%, ensuring compliance with regulatory standards and minimizing psychoactive effects [4].

Scientific research indicates that the oral bioavailability of CBD is approximately 6% when administered on an empty stomach, increasing to about 19% when consumed with fatty foods. This enhancement is likely attributable to CBD’s highly lipophilic properties, which result in limited gastric solubility [9,10,11]. Collectively, these findings indicate that the pharmacokinetics of CBD differ substantially based on the route of administration and the food matrix in both animal models and humans. A potential mechanism for the influence of food on CBD absorption involves its fat and caloric content, which may delay gastric emptying, stimulate bile secretion, and affect luminal metabolic processes. Co-administration of food has been reported to enhance the bioavailability of CBD [11]. Specifically, dosing CBD in the presence of a high-fat meal leads to improved and more predictable pharmacokinetics, which in turn could result in better pharmacodynamic outcomes, including any observed therapeutic efficacy [12]. Based on the observations about CBD bioavailability, we chose to immobilize CBD within a double emulsion gel matrix containing fats, which could improve the release of the compound in the intestine.

Following primary studies in which we determined the absence of any effect of 1% CBD additive in MRS broth on the growth and viability of probiotic bacteria we decided to add another component to the system that could help improve human health. Probiotic bacteria are associated with various health benefits. They may strengthen gut barrier integrity, enhance gastrointestinal function, improve gut barrier function, and modulate the immune system [13]. In addition, it is known that certain probiotic strains, referred to as psychobiotics, can help support mental health by acting through the gut–brain axis [14]. Building upon this foundation, we opted to incorporate *Lactiplantibacillus plantarum* DSM 24624, a well-documented probiotic strain, and CBD into the different phases of double emulsion gel as a delivery system to explore potential effects on human microbiota. By leveraging the protective properties of a double emulsion gel matrix, as it was shown in our previous studies [15], the formulation we investigate aims to optimize the survivability of probiotic bacteria as they transit through harsh gastric conditions and to facilitate the sustained release of probiotic and CBD in the colon. This integrative approach could potentially amplify the health-promoting outcomes associated with both CBD and probiotics, offering a novel strategy for modulating gut microbiota, supporting immune function, and advancing the therapeutic efficacy of functional foods. Taking these into account, our study employed a dynamic Simulator of the Human Intestinal Microbial Ecosystem (SHIME^®^) system to closely mimic human gastrointestinal conditions for detailed investigation of the interactions between probiotic bacteria *L. plantarum*, CBD, and the complex microbial communities residing within the gastrointestinal tract. This method allows comprehensive monitoring of microbiota shifts and metabolite production across distinct colon compartments. What is more, it provides valuable insight into how probiotics and CBD, when encapsulated in different phases of innovative delivery systems like double emulsion gel, may modulate gut microbial composition and activity, with particular emphasis on metabolites such as short-chain fatty acids (SCFAs) and lactates, that play a pivotal role in regulating immune responses and overall gut health. In this context, the present study was designed to evaluate the potential of the double emulsion gel system to deliver probiotics *L. plantarum* and CBD to the colon, while also determining the impact on the gut microbiota.

## 2. Materials and Methods

### 2.1. Preparation of Double Emulsion Gel Containing CBD

To prepare the bacterial suspension for encapsulation, *Lactiplantibacillus plantarum* DSM 24624 culture, bacteria from the slant De Man–Rogosa–Sharpe (MRS) agar (Biolife, Milan, Italy) were transferred to 10 mL of MRS broth (Biolife, Milan, Italy) and pre-cultured at +37 °C (primary inoculum). After 24 h of incubation, 1% of the primary inoculum was transferred to sterile MRS broth and incubated for another 20 h (secondary inoculum). Once the stationary phase of bacterial growth was reached, the medium containing the bacterial cells was centrifuged (MPW 260 RH centrifuge, MPW Med. Instruments, Warsaw, Poland) at 6000 rpm (4427× *g*) for 10 min at +4 °C. The supernatant was discarded, and the cells were suspended in sterile phosphate buffer (Oxoid, Basingstoke, UK).

The water-in-oil-in-water double emulsion with gelled oil and external water phases (W/O_gel_/W_gel_) was obtained following the method described by Laurita-Varnaitė Kapočė et al. [15]. The primary emulsion and double emulsion gel were formed at room temperature (+20  ±  2 °C) using an Ultra-Turrax rotor-stator system (IKA T-18 basic, Staufen, Germany). Firstly, primary W_1_/O emulsion was made by the slow addition of W_1_ to O at a ratio of 1:4, with subsequent homogenization (10 min, 7000 rpm). Phase W_1_ consisted of a 10^8^–10^9^ *Lactiplantibacillus plantarum* DSM cell suspension in phosphate buffer, whereas phase O was formulated by dissolving 4% (wt.) polyglycerol polyricinoleate (PGPR) (Danisco, Copenhagen, Denmark) and 10% (wt.) carnauba wax (Thermo Fisher GmbH, Kandel, Germany) in rapeseed oil (local market, Kaunas, Lithuania) heated to +90 ± 0.5 °C and then cooled to +20 ± 2 °C followed by the addition of 5% (wt. of the total double emulsion) cannabidiol (CBD isolate, >99% purity, provided by JVC Biosyyd). Further, external-phase W_2_ was prepared according to the following description. In total, 12% (wt.) of whey protein isolate Lacprodan^®^ SI-9213 (Arla Foods Ingredients, Videbaek, Denmark) was dispersed in distilled water and stirred with a magnetic stirrer at 1000 rpm (IKA C-Mag HS 7, Staufen, Germany). After 1 h of agitation, the whey protein isolate solution was heated to +100 ± 0.5 °C for 1 h in a water bath (Wisd WiseBath, WITEG Labortechnik, Wertheim, Germany). Once the solution had thickened after the given time, it was cooled to +20 ± 2 °C. In order to obtain the final W/O_gel_/W_gel_ emulsion, W_1_/O was homogenized with W_2_ at a ratio of 2:3 (5 min, 10,000 rpm).

### 2.2. Experimental Design of Dynamic Simulator of the Human Intestinal Microbial Ecosystem (SHIME^®^)

The Simulator of Human Intestinal Microbial Ecosystem (SHIME^®^) (ProDigest, Ghent, Belgium) is a computer-controlled simulator representing the human gastrointestinal tract [16]. The SHIME^®^ consists of five closed compartments that mimic different parts of the human digestive tract: the stomach (ST), small intestine (SI), and large intestine, which is divided into the ascending colon (AC), transverse colon (TC), and descending colon (DC). The large intestine compartments were modified by the addition of a mucosal compartment that contained carriers (K1 carrier, AnoxKaldnes AB, Lund, Sweden) coated with a layer of mucin-agar (SigmaAldrich Chemie GmbH, Steinheim, UK). To mimic in vivo conditions, pH, temperature, volumetric capacity, and retention time of different compartments were automatically controlled as described in the manufacturer’s proprietary protocol. Throughout the entire experiment, an anaerobic condition in the vessels was maintained by injecting nitrogen gas three times a day for 20 min.

Fecal sample. The donor for the fecal sample was chosen on the basis of the following criteria: volunteer aged 25–30 years, no use of prebiotics, probiotics, antibiotics, or other medications in the past six months, normal body weight, and no acute, chronic, or infectious diseases. In order to minimize oxygen exposure, the fecal sample was collected in a sterile, sealed container with an AnaeroGen™ sachet (Thermo Scientific, Oxoid Ltd., Hampshire, UK) and immediately transported to the laboratory. For the inoculation of colonic vessels, a 20% (wt./v) fecal inoculum was immediately prepared by suspending the sample in a sterile anaerobic phosphate buffer containing sodium thioglycolate (Sigma-Aldrich Chemie GmbH, Steinheim, UK), homogenizing (BagMixer^®^, Interscience International, Puycapel, France), and lastly by centrifuging (MPV-260R, MPW Med. Instruments, Warsaw, Poland) it at 500× *g* for 2 min.

**Figure 1:**
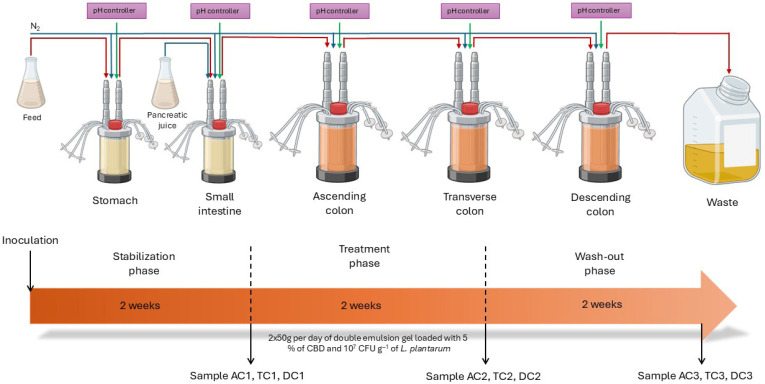
Streamlined study design and timeline overview of the SHIME^®^ experiment: AC—ascending colon, TC—transverse colon, and DC—descending colon; the numerical values correspond to experimental time points as follows: 1—after stabilization, 2—after treatment, and 3—after the wash-out phase.

SHIME^®^ run. Briefly, the SHIME^®^ experiment lasted 6 weeks: 2 weeks for start-up (stabilization), 2 weeks for treatment, and lastly, 2 weeks for washout (Figure 1). Artificial digestive juices—such as stomach juice and gastric juice—were added to the ST and SI vessels. Meanwhile, the colonic compartments were inoculated with fecal inoculum (10% *v*/*v*) together with the sterile SHIME^®^ growth medium (ProDigest, Ghent, Belgium).

During the stabilization and washout periods, the SHIME^®^ system was supplied only with the sterile SHIME^®^ growth medium (120 mL, 3 times per day) and artificial digestive juice, whereas during the treatment period, 50 g of double emulsion gel containing 5% CBD and probiotics was additionally administered twice per day in addition to the standard medium along with the 2000 U/mL of pepsin (Chemlab, Zedelgem, Belgium) and 2000 U/mL of lipase (Sigma-Aldrich Chemie GmbH, St. Louis, MO, USA) in feed and 100 U/mL of pancreatin (Sigma-Aldrich Chemie GmbH, St. Louis, MO, USA) in gastric juice. During the experiment, half of the mucin carriers were alternately replaced twice a week on the same days of the week. The mucin and lumen samples for analysis were collected after stabilization, after treatment, and after washout periods from each colonic compartment (AC, TC, and DC).

### 2.3. Microbial Community Analysis by 16S rRNA Gene Amplicon Sequencing

For microbial community analysis, DNA was extracted using the PureLink™ Microbiome DNA Purification Kit (Thermo Fisher Scientific, Pleasanton, CA, USA). This method of DNA purification is based on the triple lysis approach and PureLink spin column technology. The procedure was performed according to the manufacturer’s guidelines. The DNA of bacteria inhabiting the mucosal layer was purified from 1.2 g of mucin-agar sample collected from mucin carriers, while luminal DNA was purified from 2 mL of the sample. DNA concentration was recorded by the Qubit^®^ 3.0 fluorometer using the double-stranded DNA (dsDNA) broad range (BR) assay kit (Invitrogen, Eugene, OR, USA). The composition of microbial community was analyzed by next-generation sequencing (NGS) of V2-4 and V6-9 regions using the Ion Torrent™ Next-Generation Sequencer (Thermo Fisher Scientific, Carlsbad, CA, USA). 16S rRNA gene amplicon libraries were generated by the manufacturer’s preparation workflow using the Ion 16™ Metagenomics Kit, Ion Xpress™ Barcode, and Adapters (Thermo Fisher Scientific, Carlsbad, CA, USA). In total, 1 µL of purified DNA with a concentration of 5 ng was used to prepare DNA libraries. Barcoded libraries were purified using the Agencourt^®^ AMPure^®^ XP Reagent (Beckman Coulter, Carlsbad, CA, USA) magnetic bead-based purification. Templating and the chip loading procedures were processed automatically using the Ion Chef System with the Ion S5™ Ion 510 & Ion 520 & Ion 530 Kit—Chef (Thermo Fisher Scientific, Carlsbad, CA, USA).

### 2.4. Microbial Community Analysis by Digital Polymerase Chain Reaction

Quantification of 16S rRNA of *Enterobacteriaceae*, *Clostridium*, *Lactobacillus*, *Prevotella*, *Bacteroides* genus, and *Lactiplantibacillus plantarum* in both the mucin layer and the lumen of the SHIME^®^ system colon compartment was performed using digital polymerase chain reaction (dPCR). The concentrations of DNA samples for the dPCR were adjusted to 1000 copies of DNA/µL. In order to increase sensitivity and specificity, TaqMan^®^ minor groove binding (MGB) probes were used. The probe for the detection of the family *Enterobacteriaceae* was labeled at its 5′ ends with the fluorescent reporter dye 6-carboxy-fluorescein (FAM), while the probe for the detection of *Clostridium* was labeled with VIC^®^ (Applied Biosystems, Nieuwerkerk, The Netherlands). The probes also contained nonfluorescent quenchers (NFQ-MGB, Applied Biosystems, Nieuwerkerk, The Netherlands) attached to the 3′ ends. For the determination of *Prevotella*, *Bacteroides*, *Lactobacillus*, and *Lactiplantibacillus plantarum*, an already described probe and primer set was used. All primers and probes that have been used in this study are listed in Table 1. The 10 µL reaction mixture, containing 2 μL of Absolute Q™ DNA Digital PCR Master Mix (5X) (Thermo Fisher Scientific, Forster City, CA, USA), 1.5 μL of nuclease-free water (Applied Biosystems, St. Austin, TX, USA), 1.5 µL mixture of 1 µL each forward, reverse primers and probe, and 5 μL of DNA, was prepared.

The dPCR was performed in a total volume of 9 μL on a QuantStudio™ Absolute Q™ Digital PCR System (Thermo Fisher Scientific, Forster City, CA, USA) using its proprietary microfluidic array plate (MAP). The prepared MAP was layered with Absolute Q™ Isolation Buffer, sealed with gaskets, and subjected to initial denaturation at 95 °C for 10 min. Then, the thermal cycling protocol was carried out: cycles of denaturation at 95 °C for 15 s, then annealing and elongation at 60 °C for 60 s were repeated 40 times, followed by holding at 98 °C for 10 min. The ramp rate was 2.5 °C/s during the entire process.

### 2.5. Short-Chain Fatty Acid Analysis

In order to evaluate the metabolic activity of the gut microbial communities, quantification of short-chain fatty acids (SCFAs), such as acetate, butyrate, and propionate, was performed. SCFAs were analyzed with a Shimadzu GC-17A (Shimadzu, Kyoto, Japan) gas chromatograph equipped with an AOC-20i autosampler and coupled to a flame ionization detector. A Stabilwax-DA capillary column (30 m × 0.25 mm × 0.25 µm; Restek, PA, USA) was used. The liquid fraction of the SHIME^®^ samples from AC, TC, and DC compartments was separated by centrifugation, and the obtained supernatant was filtered by membrane filters (0.45 µm) prior to injection onto the column. Samples with a higher amount of SCFAs were diluted with 1% HCl/75% ethanol aqueous solution. Analysis conditions were as follows: column temperature was 38 °C for 3 min, then increased to 240 °C at a 15 °C/min rate and held for 3 min; injection volume was 1 µL; carrier gas was nitrogen; column flow was 0.67 mL/min; and the total run time was 20.47 min. Quantification of samples was performed by using external standards.

In addition, levels of lactate were assessed. D-lactic acid and L-lactic acid content were determined using the Megazyme Assay Kit (Megazyme, Bray, Ireland). The SHIME^®^ samples were centrifuged; supernatants were filtered by membrane filters (0.45 µm) and directly analyzed according to the kit protocol.

### 2.6. High-Performance Liquid Chromatography (HPLC) Analysis of CBD During Digestion

For the quantification of phytocannabinoid CBD, 100 mg of the freeze-dried lumen sample was accurately dispersed in 10 mL of methanol. The mixture was then agitated using a Vortex shaker for 5 min to ensure thorough extraction. Following this, the sample underwent centrifugation to separate the solid and liquid phases. The resulting supernatant was carefully filtered through a 0.22 µm membrane filter to remove particulates, and the filtrate was transferred into a 2 mL sample vial, making it ready for subsequent analysis. Analytical measurements were conducted using a Prominence-i, LC-2030C Plus high-performance liquid chromatography (HPLC) system (Shimadzu, Kyoto, Japan), equipped with an integrated pump, autosampler, and UV detector. Chromatographic separation was achieved on a NexLeaf CBX for Potency reverse phase C18 column (2.7 µm; 150 mm × 4.6 mm, Shimadzu, Japan). The gradient elution was performed using mobile phase A (0.085% orthophosphoric acid in water) and mobile phase B (0.085% orthophosphoric acid in acetonitrile). The gradient program was as follows: 70% mobile phase B from 0 to 3 min, 85% B from 3 to 7 min, 95% B from 7 to 8 min, and returning to 70% B from 8 to 10 min. The flow rate during the analysis was maintained at 1.6 mL/min, with an injection volume of 5 µL per sample. The autosampler and column temperatures were kept constant at 4 °C and 35 °C, respectively, throughout the procedure. Detection of CBD was performed at a wavelength of 220 nm to ensure accurate quantification. The concentration of CBD in the colon lumen was reported as g/L.

### 2.7. Data and Statistical Analysis

The analysis of the data obtained from the NGS was carried out by using Thermo Fisher Scientific’s integrated bioinformatics pipeline, which consists of Torrent Suite™ Software 5.18.2 for raw data transfer from the sequencer and primary analysis, and Ion Reporter™ Software 5.20 for deeper subsequent analysis. Based on the similarity, sequences were assigned into operational taxonomic units (OTUs), referred to as “counts”, and were used as an indicator of the relative abundance of different taxa on the different phylum levels in the analyzed sample. Heatmaps were generated using R software 4.5.1 with the pheatmap package. To convert into a normal distribution, data were normalized in log scale, and heatmap rows were ordered based on hierarchical clustering. The QIIME™ open-source bioinformatics pipeline was used for diversity analyses and visualizations. Alpha diversity was assessed using the Shannon Index, and statistical differences among groups were analyzed by one-way analysis of variance (ANOVA) with Tukey’s post hoc test. Bray–Curtis dissimilarity analysis was applied to assess structural differences in microbial communities across samples, and results were visualized using principal coordinate analysis (PCoA). Additionally, permutational multivariate analysis of variance (PERMANOVA) was conducted to determine statistical significance between groups. Analyses were carried out based on the data obtained from Ion Reporter™ and executed using R software version 4.5.1, incorporating the plotly, vegan, ellipse, and readr packages. Applied Biosystems QuantStudio™ Absolute Q™ Software 6.3 was used to analyze the concentration of bacterial DNA copies per µL. dPCR was conducted in technical replicate and data are presented as mean ± standard deviation. SCFAs, lactates, and CBD concentrations were measured in technical triplicate. Data are presented as mean ± standard deviation and analyzed by ANOVA with Fisher LSD test using Statistica 8.0. A *p*-value < 0.05 was considered statistically significant.

## 3. Results

### 3.1. Changes in Microbial Composition After Exposure to CBD Treatment

**Figure 2:**
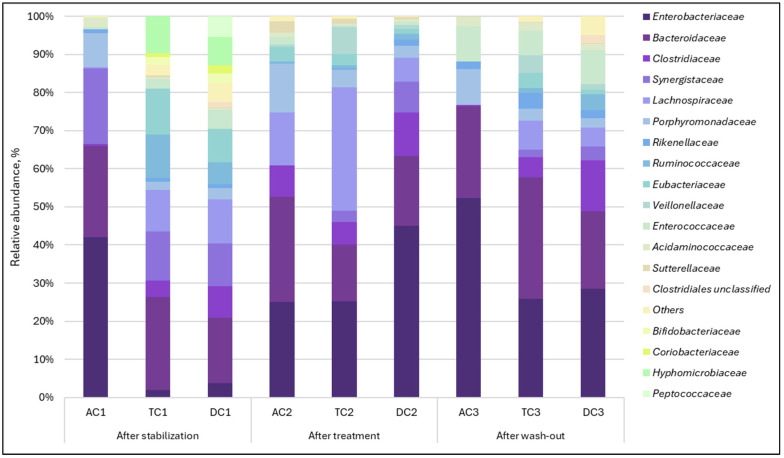
Relative abundance of gut bacteria of the mucosal phase at a family level in the different compartments of SHIME^®^ colon. AC indicates the ascending colon, TC—transverse colon, DC—descending colon; the numerical values correspond to experimental time points as follows: 1—after stabilization, 2—after treatment, and 3—after the wash-out phase.

Relative abundance. It is widely recognized that the composition of microbial communities varies among different locations of the digestive tract. Microorganisms residing on the intestinal mucosa, along with those present in the luminal contents of the intestine, constitute essential components of the microbiota [22,23]. For the above reason, we assessed the impact of supplementation with double emulsion gel containing CBD and *L. plantarum* on both gut luminal and mucosal microbial composition by analyzing the data at both family and species levels. The relative abundance of bacterial taxa within both lumen and mucin samples demonstrated variation when evaluating the abundance of specific families in response to treatment. Regarding the mucosal microbiota, 40 bacterial families were detected. In the AC compartment, it exhibited a substantial increase in the relative abundance of *Lachinospiraceae*, rising from 0.22% to 14.01% following treatment (Figure 2).

A comparable trend was noted with *Clostridiaceae*, as its relative abundance increased from 0.45% to 8.16%. Upon completion of the washout period, the abundance of the mentioned families returned to baseline levels. It was also observed that supplementation caused the cessation of the *Synergistaceae* family. After treatment, the family, previously ranked among the top three in terms of relative abundance, was no longer detected. When assessing the TC and DC compartments, a decrease in the *Synergistaceae* family was noted here as well, but unlike AC, this family remained present after treatment. The effect was more pronounced in the TC compartment, with a reduction from 12.91% to 2.99%. A major bloom of the *Enterobacteriaceae* family after treatment in the TC and DC compartments was found. The relative abundance of this family increased from 1.98% to 25.15% in the TC compartment and from 3.82% to 44.98% in the DC compartment.

**Figure 3:**
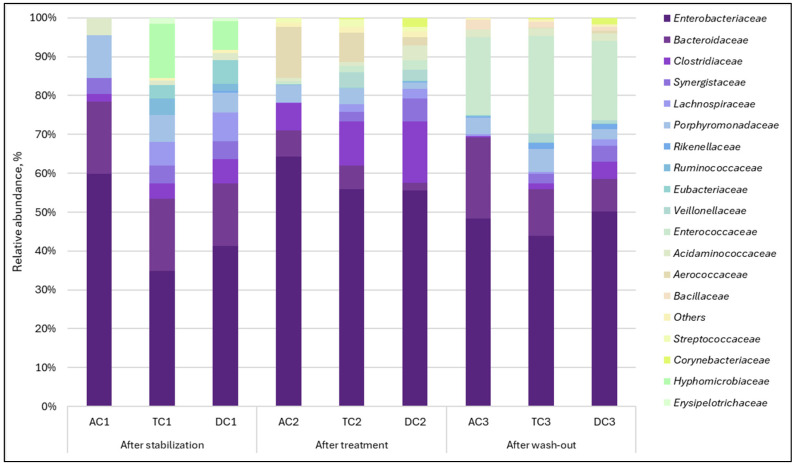
Relative abundance of gut bacteria of the lumen phase at a family level in the different compartments of the SHIME^®^ colon. AC—ascending colon, TC—transverse colon, and DC—descending colon; the numerical values correspond to experimental time points as follows: 1—after stabilization, 2—after treatment, and 3—after the wash-out phase.

Analysis of lumen samples revealed different changes from those observed in the mucin phase. In this case, 37 bacterial families were detected. The most significant alterations occurred in the relative abundance of the *Aerococcaceae*, *Bacteroidaceae*, *Clostridiaceae*, *Enterococcaceae*, and *Hyphomicrobiaceae* families (Figure 3). Bacteria of the *Aerococcaceae* family, which were undetectable after stabilization, became one of the most prominent families in the proximal colon (AC and TC). It is worth mentioning that it acted as a transient microorganism and returned to almost undetectable levels after the wash-out period. A relative increase in the *Enterococcaceae* family, which was also undetectable after the stabilization phase, was observed. However, unlike the *Aerococcaceae* family, this family accounted for a large proportion of the microbial composition after the wash-out phase.

The relative abundance of another family, *Clostridiaceae*, increased after treatment, with a more noticeable effect in the TC and DC compartments. We also observed that the examined treatment led to the suppression of *Bacteroidaceae* and *Hyphomicrobiaceae* relative abundances. When treatment was terminated (during the wash-out phase), the *Bacteroidaceae* relative abundance recovered. Meanwhile *Hyphomicrobiaceae* family was completely eliminated. Similar to observations with mucin, the lumen was primarily populated by *Enterobacteriaceae*, which exhibited an increased relative abundance during the treatment period.

The analysis of the relative operational taxonomic unit (OTU) abundance heatmap provided a detailed perspective on species-level shifts in the gut microbiota throughout different phases of the SHIME^®^ experiment. The administration of the double emulsion gel containing CBD and *L. plantarum* exhibited distinct effects on both the mucin layer and the luminal bacterial populations.

**Figure 4:**
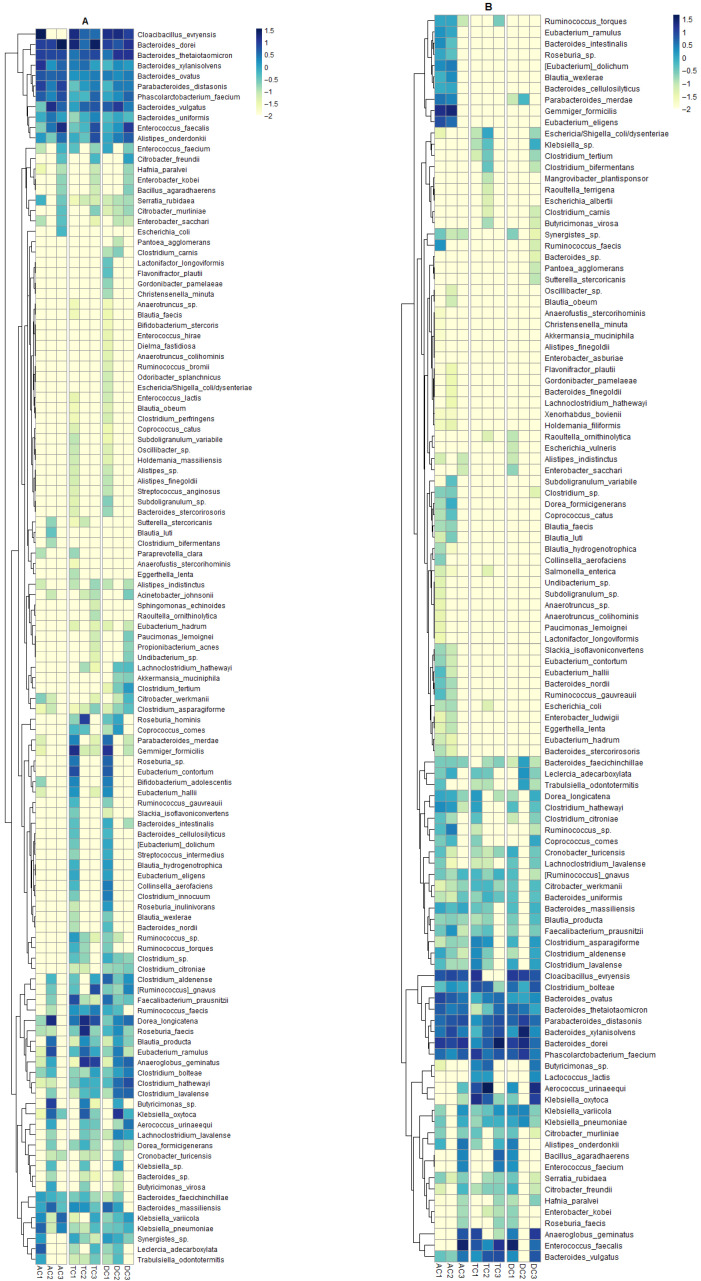
Heatmap illustrating relative abundance of gut bacteria at a species level in the different compartments of SHIME^®^ colon: (**A**)—mucin layer and (**B**)—colon lumen; AC1, TC1, and DC1—samples after stabilization; AC2, TC2, and DC2—samples after treatment; AC3, TC3, and DC3—samples after wash-out (AC—ascending colon, TC—transverse colon, and DC—descending colon).

In the mucin, the most pronounced change was observed in the *Cloacibacillus evryensis* species (Figure 4A). This bacterium accounted for nearly 34% of the relative abundance in the ascending colon (AC) compartment after stabilization. Following the CBD and probiotic treatment, *C. evryensis* was no longer detected in this compartment, and its absence persisted through the wash-out period. A notable decrease in its relative abundance was also recorded in the transverse colon (TC) compartment. In contrast, the descending colon (DC) compartment did not exhibit an immediate reduction in this species post-treatment, but a decrease became apparent after the wash-out phase. Relevant alterations were also identified among four *Bacteroides* species after treatment. The relative abundance of *Bacteroides dorei* decreased in both TC and DC compartments, returning to prior levels following wash-out. In the AC compartment, however, a substantial and sustained increase in *B. dorei* was observed after the wash-out phase, rising from approximately 9% to 35.4%. On top of that, treatment with the CBD and probiotic emulsion led to increased relative abundances of *Bacteroides thetaiotaomicron* and *Bacteroides vulgatus*, while *Bacteroides xylanosolvens* showed reduced abundance within the AC compartment. Further notable changes included an increase in the relative abundance of *Dorea longicatena* from 0.15% to 18% in the AC compartment and from 2.82% to 16.04% in the TC compartment after treatment. A similar pattern was observed for *Klebsiella oxycota*, only this time the DC compartment experienced a marked rise from undetectable levels to 13.05% after treatment. A group of bacteria from the *Roseburia* genus was also impacted. The relative abundance of *Roseburia faecis* and *Roseburia hominis* increased under the influence of CBD and probiotics, with this effect being particularly noticeable in the TC compartment. Furthermore, more relative abundance of species changes was found in the TC compartment. For example, after treatment, *Anaeroglobus geminatus* increased significantly. In contrast, species such as *Parabacteroides merdae*, *Eubacterium contortum*, and *Collinsella aerofaciens* experienced decreases in relative abundance, with some disappearing altogether.

The data obtained from microbiome analysis of the lumen also reveals some interesting patterns in the distribution of species (Figure 4B). For instance, *Aerococcus urinaeequi* exhibited a major shift in relative abundance. This observation is noteworthy, as the bacteria were nearly undetectable within the AC compartment. By contrast, the TC compartment exhibited a marked increase in relative abundance, rising from 6.97% post-stabilization to 43.847% following treatment. In addition, the DC compartment demonstrated a distinct trend, with an elevated relative abundance observed after the washout phase. After supplementation of the SHIME^®^ system with double emulsion gel, the relative abundance of several bacterial species increased, including *Bacteroides vulgatus*, *Bacteroides xylanisolvens*, *Blautia wexlerae*, *Butyricimonas* spp., *Faecalibacterium prausnitzii*, *Gemmiger formicilis*, and *Parabacteroides distasonis*. While the negative effect of this supplementation on relative abundance, although not consistently across all colon compartments, was found in *Anaeroglobus geminatus*, *Cloacibacillus evryensis*, *Eubacterium eligens*, and *Klebsiella oxytoca*. Very interesting findings emerged from the OTU analysis of *Enterococcus faecalis* and *Phascolarctobacterium faecium*. In the AC compartment, the relative abundance of *E. faecalis* remained extremely low—below 0.1%—during both the stabilization and treatment periods. However, a dramatic increase was observed following the wash-out phase, with the relative abundance surging to 30.15%. In the TC compartment, the relative abundance of *E. faecalis* initially decreased from 6.9% after stabilization to 1.98% after treatment. After the wash-out period, the abundance rose again, reaching 15.54%. Even stranger things happened in the DC compartment—after stabilization, the relative abundance of *E. faecalis* was the highest among all species and reached 31.19%. It was no longer detected after treatment, but was found again at 3.72% after the wash-out phase.

Another culture that also yielded intriguing results was *Phascolarctobacterium faecium*. The relative abundance of this culture within the AC compartment remained almost consistent throughout the experiment. Meanwhile, in the TC compartment, the relative abundance decreased from 11.87% to 3.01% after two weeks of exposure to the double emulsion. The DC compartment displayed the opposite effect, where the relative abundance increased from 5.66% after stabilization to 13.83% post-treatment. Together, these findings underscore the complex and compartment-specific responses of gut microbial species to dietary supplementation and subsequent wash-out, emphasizing the dynamic nature of microbial community structure within the simulated colon environment.

After analysis of relative abundance, diversity indices were subsequently employed to describe the compositional complexity of a single sample, as well as between samples.

**Figure 5:**
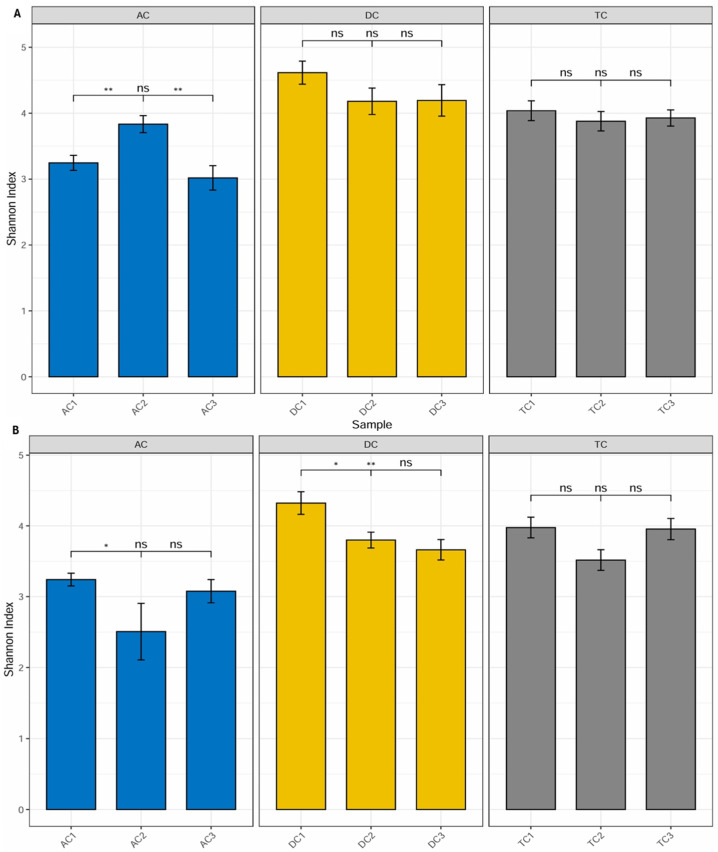
Shannon Diversity Index in the different compartments of the SHIME^®^ colon: (**A**)—mucin phase and (**B**)—lumen phase (*—*p* < 0.05; **—*p* < 0.01). AC—ascending colon, TC—transverse colon, and DC—descending colon; the numerical values correspond to experimental time points as follows: 1—after stabilization, 2—after treatment, and 3—after the wash-out phase. In contrast to the positive effect observed in the mucin layer, analysis of the luminal microbial communities revealed more pronounced differences in alpha diversity following treatment (**B**). Specifically, the Shannon Diversity Index in the lumen of the ascending colon (AC) demonstrated a statistically significant decrease (*p* < 0.05) after treatment compared to levels measured after the stabilization phase. This reduction in alpha diversity indicates a decline in the compositional complexity of the microbial community within this compartment as a result of the intervention. A similar trend was detected in the descending colon (DC) compartment, where the Shannon Diversity Index also exhibited a statistically significant decrease (*p* < 0.05) after treatment. Notably, this effect persisted even after the washout period, with the Shannon Index remaining significantly lower (*p* < 0.01) when compared to values recorded following stabilization. These findings suggest that the reduction in alpha diversity induced by the treatment in the lumen phase of both AC and DC compartments was not immediately reversible upon cessation of supplementation.

Diversity. Alpha diversity is generally described as an indicator of the compositional complexity of a community within a specific site. Alpha diversity tends to rise with both the number of species present and the evenness of their relative abundances. For the analysis of alpha diversity, we calculated the Shannon Diversity Index, which takes into account both species richness and evenness. Statistically significant differences (*p* < 0.01) were observed between mucin samples AC1 (after stabilization) and AC2 (after treatment), as well as between AC2 and AC3 (after wash-out) (Figure 5A). The data indicate an increase in the Shannon index, and the observed differences imply that the applied treatment has affected alpha diversity. Furthermore, the lack of significant differences between AC1 and AC3 indicates that the effect on microbiota diversity was transient. No statistically significant differences were identified in the remaining compartments.

**Figure 6:**
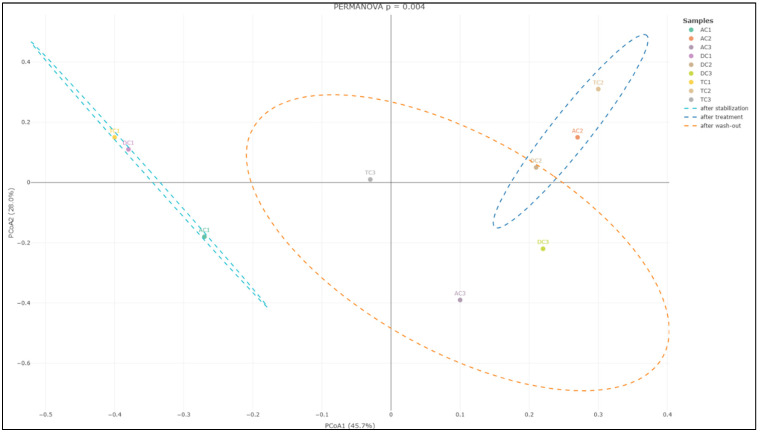
PCoA of Bray–Curtis dissimilarity (beta diversity) with PERANOVA analysis in the mucin phase. AC—ascending colon, TC—transverse colon, and DC—descending colon; the numerical values correspond to experimental time points as follows: 1—after stabilization, 2—after treatment, and 3—after the wash-out phase. Ellipses represent individual samples in the mucin-after-stabilization (sky-blue color), mucin-after-treatment (blue), or mucin-after-wash-out phase (orange) groups.

In addition to evaluating alpha diversity, the study employed Bray–Curtis analysis to examine the similarities and differences among gut microbial communities, providing insights into beta diversity. Beta diversity is a crucial metric in microbiota research, as it measures the taxonomic variation between distinct sample groups, enabling a deeper understanding of how microbial compositions shift in response to treatment with the double emulsion gel with CBD and *L. plantarum*. The Bray–Curtis analysis of bacterial composition profiles revealed statistically significant differences among the mucin sample groups collected after stabilization, following treatment, and after the wash-out phase (*p* = 0.004; Figure 6). This finding indicates that the treatment administered had a notable impact on the structure of the mucin-associated microbiota, with each phase presenting a distinct microbial profile.

**Figure 7:**
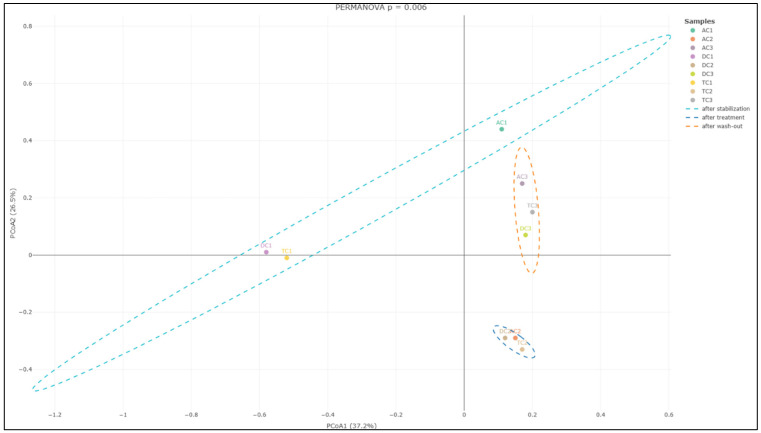
PCoA of Bray–Curtis dissimilarity (β-diversity) with PERANOVA analysis in the colon lumen. AC—ascending colon, TC—transverse colon, and DC—descending colon; the numerical values correspond to experimental time points as follows: 1—after stabilization, 2—after treatment, and 3—after the wash-out phase. Ellipses represent individual samples in the lumen-after-stabilization (sky-blue color), lumen-after-treatment (blue), or lumen-after-wash-out phase (orange) groups.

Further analysis focusing on the colon lumen (Figure 7) demonstrated that the structure of the gut microbial community underwent significant changes across the different experimental phases. Statistically significant alterations were observed in the community composition (*p* = 0.006) when comparing the stabilization, treatment, and wash-out periods. Principal component analysis further supported these findings, identifying two principal component scores that account for 37.2% and 26.5% of the total variation. These components highlight substantial shifts in microbial composition associated with each phase of the experiment.

**Figure 8:**
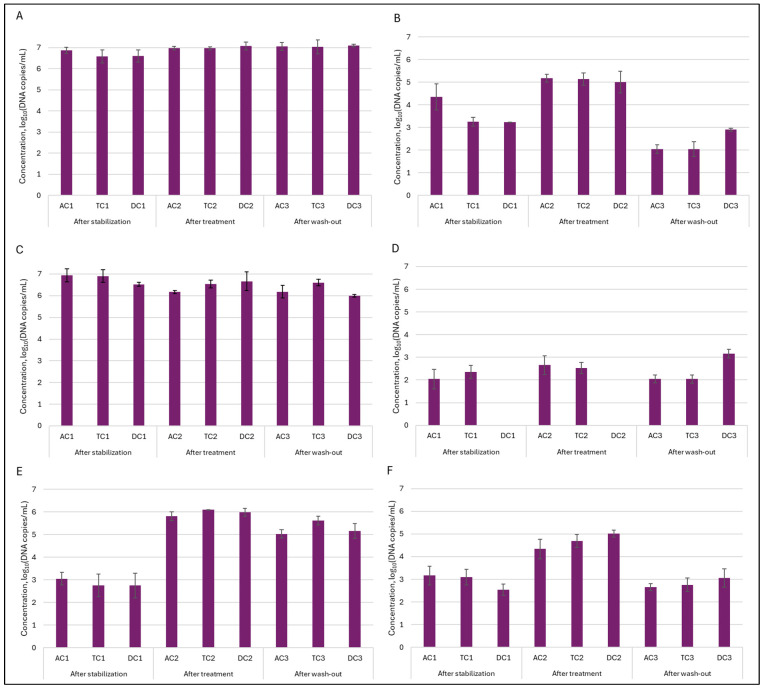
Variation in absolute levels (log_10_DNA copies/mL) of six different taxonomic groups in the lumen during SHIME^®^ run: (**A**)—*Enterobacteriaceae*; (**B**)—*Clostridium*; (**C**)—*Bacteroides*; (**D**)—*Prevotella*; (**E**)—*Lactobacillus*; and (**F**)—*Lactiplantibacillus plantarum.* AC—ascending colon, TC—transverse colon, and DC—descending colon; the numerical values correspond to experimental time points as follows: 1—after stabilization, 2—after treatment, and 3—after the wash-out phase.

Quantitative dPCR. In order to evaluate quantitative alterations in the gut microbiota, dPCR analysis was employed. The most abundant family of *Enterobacteriaceae* and genus of *Bacteroides* were selected as targets for further investigation. In addition, *Clostridium* and *Prevotella* were also included in the analysis. This strategic selection allowed for more comprehensive monitoring of key bacterial groups known to play a significant role in the gut microbiota. For the assessment of the double emulsion gel system’s ability to release encapsulated *L. plantarum*, genus *Lactobacillus* and species *L. plantarum* were also selected as a target microorganism. Analysis of the lumen indicated (Figure 8) that *Enterobacteriaceae* and *Bacteroides* were present in the highest concentrations within the gut microbiota, reaching levels of up to 7 log_10_ DNA copies/mL. These findings are consistent with the DNA sequencing results observed. While absolute levels of *Enterobacteriaceae* remained similar, NGS results indicated an increase in the relative abundance of this family. This suggests that the observed increase in relative abundance was not due to a proliferation of *Enterobacteriaceae* themselves, but rather due to a decrease in the abundance of other bacterial families. Administration of the double emulsion gel with *L. plantarum* and CBD resulted in a decrease in *Bacteroides* values; however, the concentration remained within the range of 6.17–6.66 log_10_DNA copies/mL. A more substantial effect was noted upon the quantification of *Clostridium*. The concentration following stabilization was 4.35 ± 0.58 log_10_DNA copies/mL, 3.26 ± 0.19, and 3.24 ± 0.01 log_10_DNA copies/mL in AC, TC, and DC compartments, respectively. After treatment, the amount of *Clostridium* in the lumen increased similarly in all compartments, with the value around 5–5.17 log_10_DNA copies/mL. Again, this observation was corroborated by analysis at the bacterial family level. Another investigated genus—*Prevotella*—exhibited a distinct pattern. During both the stabilization and treatment phases, its abundance in the AC and TC compartments was similar, whereas it was not detected in DC. However, after the wash-out phase, the level of *Prevotella* peaked in the DC compartment, attaining 3.17 ± 0.19 log_10_DNA copies/mL—the highest among all colon segments assessed.

**Figure 9:**
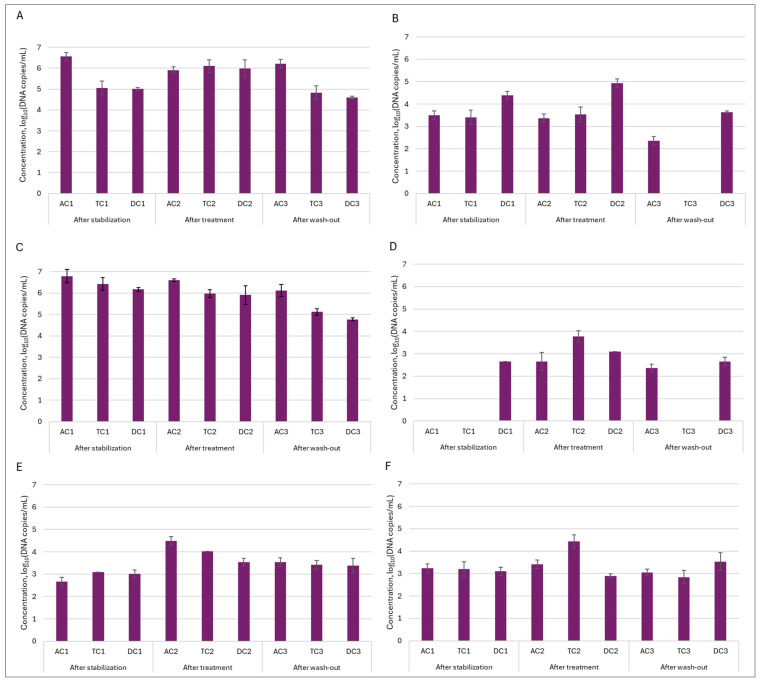
Variation in absolute levels (log_10_DNA copies/mL) of six different taxonomic groups in mucin during SHIME^®^ run: (**A**)—*Enterobacteriaceae*; (**B**)—*Clostridium*; (**C**)—*Bacteroides*; (**D**)—*Prevotella*; (**E**)—*Lactobacillus*; and (**F**)—*Lactiplantibacillus plantarum.* AC—ascending colon, TC—transverse colon, and DC—descending colon; the numerical values correspond to experimental time points as follows: 1—after stabilization, 2—after treatment, and 3—after wash-out phase.

In the mucin layer, as expected based on 16S rRNA sequencing analysis, *Enterobacteriaceae* and *Bacteroides* were the most prevalent (Figure 9). Treatment with double emulsion gel caused a minor shift in the abundance of the mentioned genus. In case of *Enterobacteriaceae*, the amount of bacteria returned to baseline, meanwhile level of *Bacteroides* continued to decrease through experimental phases, with the lowest amount of 5.11 ± 0.14 and 4.77 ± 0.06 log_10_DNA copies/mL in TC and DC compartments, respectively. The positive effect of the treatment was noted on the abundance of *Prevotella* in the mucin layer. It can be seen that after treatment amount of this genus of bacteria from an undetectable level rose to 2.65 ± 0.05 log_10_DNA copies/mL in the AC compartment and 3.78 ± 0.25 log_10_DNA copies/mL in the TC compartment.

Next, we wanted to determine how the treatment affected the quantity of *Lactobacillus* genus bacteria—one of the most thoroughly researched probiotic genera from a scientific perspective. This analysis was very important in determining whether our double emulsion gel system effectively delivered and released immobilized *L. plantarum* bacteria during digestion. The obtained results indicated that the effect was major and, in all instances, the number of bacteria of this genus in the colon lumen increased from approx. 2.76–3 log_10_DNA copies/mL to approx. 4.3–5 log_10_DNA copies/mL after treatment and remained elevated after the wash-out phase. Meanwhile, in the mucin layer, such an effect was not so prominent. Then we delved deeper into the genus of *Lactobacillus* and conducted an analysis on the presence of *Lactiplantibacillus plantarum*, and we saw the same tendency, as with the *Lactobacillus* genus. Only in this instance, following the increase observed after the treatment phase, the quantity of *L. plantarum* in the lumen returned to a baseline level after was-out phase. These results confirmed that the emulsion was effective at releasing the encapsulated probiotic bacteria.

### 3.2. Metabolic Activity of Microbial Communities

**Figure 10:**
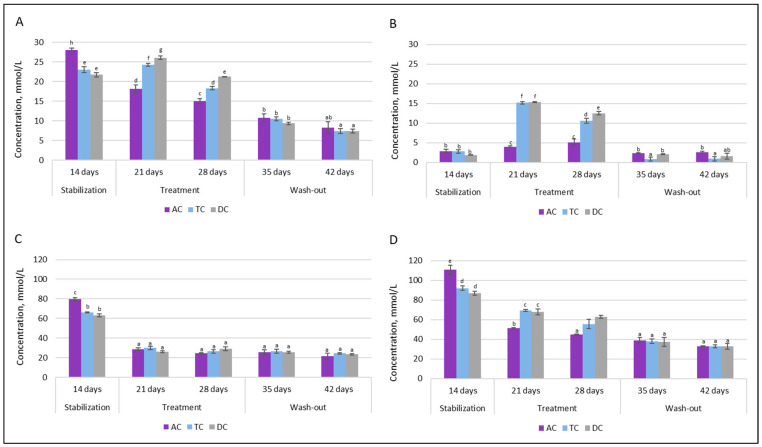
Changes in SCFAs levels after stabilization (14 days after initial system inoculation), during treatment (21–28 days) and wash-out (35–42 days) phases: (**A**)—propionate; (**B**)—butyrate; (**C**)—acetate; and (**D**)—total level of SCFAs (AC—ascending colon, TC—transverse colon, and DC—descending colon). Results marked with different lowercase letters are statistically significantly different (*p* < 0.05).

SCFAs analysis was employed to characterize the composition of intestinal microbial communities in terms of metabolic activity. Figure 10 depicts the levels of acetate, butyrate, propionate, and total SCFAs (sum of acetate, butyrate, and propionate) after stabilization (14 days after the initial SHIME^®^ inoculation) and during treatment (21–28 days) and wash-out (35–42 days) phases.

It can be seen that the intervention of the double emulsion gel loaded with CBD and probiotics significantly affected acetate levels in all of the colon compartments after the first week. The concentration decreased from 79.70 mmol/L to 28.95 mmol/L in the AC compartment, from 66.20 mmol/L to 29.99 mmol/L in the TC compartment, and from 63.36 mmol/L to 26.36 mmol/L in the DC compartment. After an additional week of treatment and throughout the wash-out phase, the concentration of acetate stayed similar in each colonic vessel.

As opposed to acetate, a positive metabolic impact was rapidly observed with increased SCFAs synthesis towards butyrate. Following one week of supplementation, there was a substantial increase in TC and DC vessel concentrations, rising from approximately 3 mmol/L to about 15 mmol/L, whereas the concentration in the AC vessel remained similar to the level observed after stabilization. A comparable trend was noted following the second week of supplementation. However, the concentration of total SCFAs remained at lower levels during treatment with double emulsion gel loaded with CBD and *L. plantarum*. This was not only due to a significant drop in acetate concentration, but also to a reduction in propionate level. During the washout phase, when only the basal medium without the test supplement was supplied to the system, the butyrate level returned to the concentration prior to treatment (~1–3 mmol/L), while the propionate concentration continued to decrease throughout this phase, resulting in a twofold decrease in the total amount of SCFAs.

**Figure 11:**
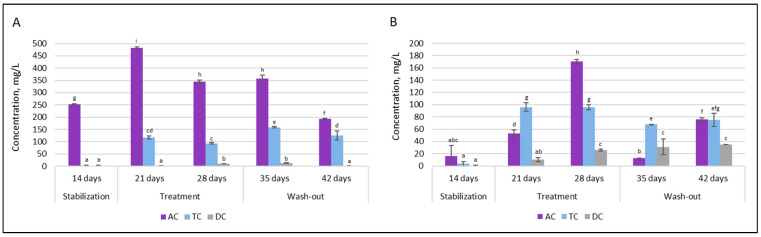
Changes in lactate levels after stabilization (14 days after initial system inoculation) during treatment (21–28 days) and wash-out (35–42 days) phases: (**A**)—D-lactic acid and (**B**)—L-lactic acid (AC–ascending colon, TC—transverse colon, and DC—descending colon). Results marked with different lowercase letters are statistically significantly different (*p* < 0.05).

The levels of another metabolic marker—lactate—are presented in Figure 11.

Upon double emulsion gel loaded with probiotics and CBD treatment, the production of D-lactic acid in the proximal colon was increased significantly, from 252.5 ± 2.12 mg/L to 482.5 ± 3.53 mg/L in AC and from 1.5 ± 0.12 mg/L to 117.5 ± 6.36 mg/L in TC. In contrast, such an effect was not prominent in the distal part of the colon. Under all conditions, production of D-lactic acid in the DC compartment was very low. The levels of L-lactic acid also increased after treatment. As with D-lactic acid, there was an overall tendency for treatment to increase levels of L-lactic acid in the proximal colon.

### 3.3. Release of CBD from Double Emulsion Gel During Digestion in SHIME^®^

**Figure 12:**
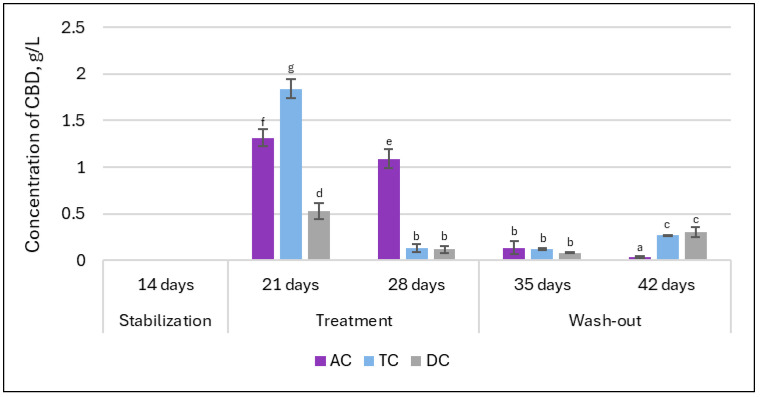
Concentration of CBD in the lumen in the different compartments of SHIME^®^ (AC—ascending colon, TC—transverse colon, and DC—descending colon). Results marked with different lowercase letters are statistically significantly different (*p* < 0.05).

Our previous research on double emulsion gel loaded with *L. plantarum* demonstrated that the system was able to protect encapsulated bioactive materials from environmental conditions and during static in vitro digestion [15]. However, it remains necessary to determine whether the system will demonstrate sufficient efficacy in releasing the encapsulated materials. dPCR analysis indicated the potential for successful release of *L. plantarum*. In the next step, HPLC analysis was employed to evaluate whether CBD is released from the double emulsion gel during digestion. The concentrations of CBD in lumen samples throughout the experimental timeline are presented in Figure 12. As anticipated, no CBD was detected in any samples collected following the stabilization period, confirming the absence of baseline CBD prior to treatment initiation. After the first week of administration of double emulsion gel loaded with CBD, the amount detected in proximal (AC and TC compartments) colon samples was at the highest levels during the whole experiment (1.3 ± 0.09 g/L and 1.84 ± 0.1 g/L, respectively). However, after an additional week of continuous treatment, the highest decrease in CBD levels in the TC compartment was evident (to 0.13 ± 0.04 g/L).

One week after termination of supplementation (first week of the wash-out phase), the concentrations of CBD measured across TC and DC compartments closely resembled those found at the end of the second week of treatment. Interestingly, by the end of the second week of the wash-out phase, CBD levels in the TC and DC compartments showed an increase, suggesting a potential delayed release or redistribution of CBD within these regions subsequent to the termination of supplementation.

## 4. Discussion

When we analyzed microbiota composition changes, it became clear that the double emulsion gel with *L. plantarum* and CBD had a different effect on the bacterial communities present in the mucus and lumen. The first observation was a marked increase in the relative abundance of the *Lachinospiraceae* family in the mucin. The predominant genera identified in the human gut microbiota include *Blautia*, *Coprococcus*, *Dorea*, *Lachnospira*, *Oribacterium*, *Roseburia*, and *Ruminococcus* [24]. Recent research suggests that *Lachnospiraceae* may contribute significantly to human health, with analyses of human gut metagenomic datasets revealing that this taxon constitutes approximately 10% of the total healthy gut microbiome [25,26]. Controversially, although species within the *Lachinospiraceae* family are typically efficient producers of short-chain fatty acids (SCFAs) [27], a marked increase in their abundance did not correspond with SCFA levels in the AC compartment; instead, a reduction in those levels was observed. More in-depth analysis of mucin at strains level revealed an increase in the *Roseburia* genus, members of the aforementioned family, specifically *R. faecis* and *R. hominis*. During carbohydrate metabolism, *Roseburia* strains tend to consume acetate while increasing butyrate production, which supports the idea that butyrate is crucial in facilitating interactions between *Roseburia* and its host [28], is very important for the control of inflammatory processes [29], and may help explain the reduction of acetate levels. Since in this instance, the substantial increase in these species was observed primarily in the TC compartment, this observation coincides with the increase in butyrate and decrease in acetate after treatment. The concentration of butyrate was significantly elevated compared to post-stabilization levels. This is a significant observation concerning metabolic activity, as butyrate functions as a primary energy source for colonocyte cells [30]. It strengthens the intestinal barrier by modulating the expression of tight junction proteins [31], promotes intestinal motility through the induction of gut satiety hormone peptide YY [32], reduces oxidative stress [33,34], and possesses anti-inflammatory effects [30,35]. The relative abundance of *Roseburia* in the colonic lumen was very low, revealing that the mucosal layer was a preferable environment for colonization. This observation corresponds with data obtained by other scientists [36]. What is more, the increase in relative abundance of the *Lachinospiraceae* family in mucin may have been caused by a relative increase in the *Dorea longicatena* species. This bacterial culture is positively associated with appendicular lean body mass in several studies [37,38]. In another interesting study, authors Prudêncio et al. [39] concluded that *Dorea longicatena*, along with red meat intake, was associated with the improvement of insulin resistance after gastric bypass surgery.

Furthermore, an increase in relative abundance after exposure to double emulsion gel with probiotics and CBD was noted with the *Clostridiaceae* family, in both mucin and lumen samples. This alteration in *Clostridiaceae* quantity was further validated by dPCR analysis, which demonstrated elevated levels of *Clostridium* after treatment. Some species within the *Clostridiaceae* family, such as *Clostridium perfringens* and *Clostridioides difficile*, are known for their pathogenic properties. However, most *Clostridiaceae* species found in the gastrointestinal tract maintain a commensal association with the host [40]. Given that the ability to produce butyrate is widely distributed among Gram-positive anaerobic obligates, such as *Clostridiaceae* [41], this shift, together with an increase in *Roseburia*, may account for the increase in butyrate levels observed.

Another finding was a substantial increase in the abundance of the *Enterobacteriaceae* family following treatment in both the TC and DC compartments. Quantitative analysis of *Enterobacteriaceae* indicated no notable increase in the absolute abundance of this family. Consequently, the observed relative rise may be attributed to a reduction in the abundance of other bacterial families. Although *Enterobacteriaceae* are commonly identified in most individuals, their ecological functions within the human gut remain largely unclear. Some studies suggest that this family may play a role in maintaining an anaerobic environment in the gut [42], producing vitamins [43,44], and providing protection against pathogen colonization [45]. Conversely, there are studies claiming that *Enterobacteriaceae* bloom is associated with certain diseases and may serve as a marker of intestinal dysbiosis and diseases [46,47]. *Proteobacteria*/*Enterobacteriaceae* typically constitute less than 5% of the gut microbiota in healthy individuals but may increase to 10–90% in humans with various diseases [42]. However, before concluding whether such an impact of applied treatment on microbiota is positive or negative, it is necessary to answer the question of whether *Enterobacteriaceae* blooming during specific illness is a cause or a consequence.

What is more, supplementation also led to the disappearance of the *Synergistaceae* family in some of the compartments. *Synergistaceae* are a prevalent group of bacteria within the human gut microbiota [48]. However, the existing studies lack comprehensive descriptions of this microbial family in detail; therefore, a more in-depth analysis of species is required to assess the effect of emulsions with probiotics and CBD on microbiota. The most significant alteration of species belonging to the *Synergistaceae* family was *C. evryensis*. During the digestion process in SHIME^®^, this culture exhibited a predominant relative abundance within the AC compartment. Following treatment, the culture became undetectable. In the TC compartment, the effect was less pronounced, while in the DC compartment, it was minimal. These observations suggest that the influence of the probiotic CBD emulsion varied according to the stage of digestion. Although *C. evryensis* has not been extensively characterized, multiple studies have classified this culture as pathogenic and associated with bacteremia [49,50]. What is more, *C. evryensis* has been identified as a mucin-degrading bacterium [51], and excessive proliferation of mucus degraders has been implicated in leaky gut-induced inflammation [52].

Among the families whose relative abundance was negatively affected, the *Hyphomicrobiaceae* family is worth mentioning. Usually, the *Hyphomicrobiaceae* family does not constitute a large proportion of the relative abundance [53]; in our study, the abundance of this family after stabilization in the lumen was relatively high (14.05%) in the AC compartment (Figure 3). Therefore, this change may reflect the positive effect of treatment with probiotics and CBD in restoring the homeostasis of the intestinal microbiota.

The subsequent phase involved investigating whether supplementation with double emulsion gel containing probiotics and CBD is associated with gut microbial diversity. Microbial diversity within a particular body habitat refers to both the number and distribution of distinct types of microorganisms present [54]. Alterations in gut microbial diversity have been associated with a range of human diseases; notably, diminished diversity within the gut microbiome has been linked to conditions such as obesity and inflammatory bowel disease [55]. Following treatment, a significant increase in the Shannon Index—an indicator of alpha diversity—was observed in the mucin phase within the AC compartment. Conversely, the alpha diversity of lumen communities was adversely affected by exposure to the emulsion loaded with *L. plantarum* and CBD. This observation substantiates the hypothesis that the bloom of the *Enterobacteriaceae* family after treatment resulted from a reduction in the relative abundance of other bacterial families. When assessing beta diversity, the distinctions between the three sample groups—stabilization, treatment, and wash-out—were particularly pronounced, underscoring the significant impact of the treatment on beta diversity within the colon lumen. This demonstrates that the treatment led to marked changes in the overall structure and diversity of the gut microbiota throughout the experimental timeline. To the best of our knowledge, this study is the first to evaluate the diversity of human gut microbiota communities in vitro in relation to the impact of CBD and probiotics.

An additional observation concerning the metabolic patterns of microbial communities is that, upon evaluating the concentration ratios of SCFAs, the applied treatment altered the proportions from 66:22:2.5 to 56:39:22 (acetate:propionate:butyrate) within both TC and DC compartments. The ratio of SCFAs in a healthy colon is estimated to be approximately 60:25:15 [30]. Although there was a decrease in acetate concentration after treatment, this ratio has improved, but further rigorous studies are necessary to determine how this change affects the overall health of the organism. An elevation in butyrate production is acknowledged as advantageous to human health [56]. Consequently, the observed increase in butyrate with double emulsion loaded with probiotics and CBD treatment suggests the potential of this innovative product to contribute to improving human intestinal health. In addition, the decrease in acetate and total SCFAs may be the result of alterations in microbial production or shifts in microbial cross-feeding dynamics. However, the biological relevance of these in vitro findings remains undetermined in the absence of human studies. Another noteworthy finding from the metabolite analysis was the increase in lactate concentrations following treatment. While the increase in L-lactic acid may contribute positively to gut health, the concurrent elevation of D-lactic acid warrants caution and should be carefully monitored, particularly in vulnerable populations.

Quantitative analysis of *L. plantarum* indicated that probiotic bacteria were effectively released from the double emulsion gel, as evidenced by significantly increased counts following treatment. These results align with the observed trend in total *Lactobacillus* abundance. Importantly, the ability of *Lactobacillus* to persist in elevated levels after treatment despite dynamic gut conditions suggests a promising approach for targeted probiotic supplementation, offering valuable insights for future studies aiming to optimize microbial therapies and enhance gut health through advanced delivery systems. In addition, the increase in L- and D-lactic acid levels contributed to the confirmation that the double emulsion gel system effectively delivered *L. plantarum* bacteria, which exhibit the ability to produce lactates, to the large intestine.

Consequently, the final stage of our study focused on evaluating the concentration of CBD in the colon lumen to determine whether the double emulsion gel effectively facilitated CBD release during digestion. It was evident that CBD was released from the double emulsion gel, with significantly higher concentrations observed in the proximal colon compared to the distal part. The decrease in concentration of CBD during the second week of treatment aligns with the dynamic shifts in gut microbiota composition documented during treatment, suggesting that microbial adaptation and metabolic activity may influence the availability and persistence of CBD within the colon. Scientists Stasiłowicz-Krzemień et al. [57] found that cannabis extracts (containing 6.04 ± 0.08 mg/g plant material of CBD) increased the proliferation of lactic acid bacteria and may be beneficial for restoring the intestinal microbiota. This extract had the highest concentration of cannabinoids among those tested, and only it showed this positive effect on lactic acid bacteria growth. Although the mechanism of action was unclear, it can be assumed that lactic acid bacteria were able to adapt and metabolize the cannabis extract. This observation may account for the reduction in CBD concentration in the lumen observed during the second week of treatment in our experiment. In addition, findings after the wash-out phase underscore the dynamic behavior of CBD within the SHIME^®^ system, highlighting not only the immediate release of the compound following supplementation with the double emulsion gel, but also the possibility of sustained or delayed liberation during the wash-out phase.

Overall, administration of the double emulsion gel containing CBD and *L. plantarum* resulted in significant changes in gut microbiota composition. These variations demonstrate the complex and specific responses of bacterial communities to the experimental supplementation of probiotics and CBD-containing double emulsion gel. The data demonstrates the diversity and adaptive capacity of different species in different samples. Understanding this information sheds light on the understanding of ecological dynamics and potential interspecies interactions across diverse environments.

## 5. Conclusions

In conclusion, the analysis indicates that the double emulsion gel system utilized in this study can be adapted for the delivery and successful release of probiotics and CBD to the gastrointestinal tract. In addition, the study demonstrates that administration of a CBD and probiotics-loaded double emulsion gel in the SHIME^®^ model leads to significant changes in gut microbial community composition and metabolic activity, including increased butyrate and lactate production and shifts in bacterial families associated with health and disease. Nevertheless, the in vitro SHIME^®^ model, though valuable for studying microbial interactions and gut dynamics, has notable limitations compared to clinical trials. It lacks a physiological host environment, including the gut epithelium, immune system, stress responses, antibodies, antimicrobial agents, and dietary or genetic variability. As such, it only reveals direct microbial metabolic changes. Despite this, SHIME^®^ may serve as a non-invasive tool to assess donor–recipient compatibility through colonization success and metabolite changes. Future research should clarify CBD’s molecular actions in the human gut in vivo and include rigorous clinical trials to fully evaluate its therapeutic potential for gastrointestinal disorders.

## Figures and Tables

**Table 1:** Primers and probes used in dPCR analysis.

Target Bacteria	Primers and Probes	Oligonucleotide Sequence (5′→3′)	Amplicon Length (bp)	Reference
*Lactiplantibacillus plantarum*	Primer F	TGGATCACCTCCTTTCTAAGGAAT	144	[17]
	Primer R	TGTTCTCGGTTTCATTATGAA AAAATA		
	Probe	ACATTCTTCGAAACTTTGT		
*Clostridium* group	Primer F	CATGCAAGTCGAGCGAKG	117–123	[18]
	Primer R	TATGCGGTATTAATCTYCCTTT		
	Probe	CCCACGTGTTACTCACCCGTCCG		
*Enterobacteriaceae* group	Primer F	ATCTGGAGGAATACCGGTGG	359	[19]
	Primer R	CAACATTTCACAACACGAGCTG		
	Probe	CGTGGCTTCCGGAGCTAACGCGT		
*Lactobacillus* group	Primer F	TGGATGCCTTGGCACTAGGA	92	[17]
	Primer R	AAATCTCCGGATCAAAGCTTACTTAT		
	Probe	TATTAGTTCCGTCCTTCATC		
*Prevotella* group	Primer F	CGAACAGGATTAGATACCC	134	[20]
	Primer R	CTTTGAGTTTCACCGTTG		
	Probe	AAACGATGGATGCCCGC		
*Bacteroides* group	Primer F	GGGTTTAAAGGGAGCGTAGG	116	[21]
	Primer R	CTACACCACGAATTCCGCCT		
	Probe	TAAGTCAGTTGTGAAAGTTTGCGGCTC		

## Data Availability

The original contributions presented in this study are included in the article. Further inquiries can be directed to the corresponding author.
